# Network-based integrative multi-omics approach reveals biosignatures specific to COVID-19 disease phases

**DOI:** 10.3389/fmolb.2024.1393240

**Published:** 2024-07-08

**Authors:** Francis E. Agamah, Thomas H. A. Ederveen, Michelle Skelton, Darren P. Martin, Emile R. Chimusa, Peter A. C. ’t Hoen

**Affiliations:** ^1^ Computational Biology Division, Department of Integrative Biomedical Sciences, Institute of Infectious Disease and Molecular Medicine, Faculty of Health Sciences, University of Cape Town, Cape Town, South Africa; ^2^ Department of Medical BioSciences, Radboud University Medical Center Nijmegen, Nijmegen, Netherlands; ^3^ Department of Applied Science, Faculty of Health and Life Sciences, Northumbria University, Newcastle, United Kingdom

**Keywords:** COVID-19, multi-omics, biosignatures, random walk with restart, multi-layer networks

## Abstract

**Background:**

COVID-19 disease is characterized by a spectrum of disease phases (mild, moderate, and severe). Each disease phase is marked by changes in omics profiles with corresponding changes in the expression of features (biosignatures). However, integrative analysis of multiple omics data from different experiments across studies to investigate biosignatures at various disease phases is limited. Exploring an integrative multi-omics profile analysis through a network approach could be used to determine biosignatures associated with specific disease phases and enable the examination of the relationships between the biosignatures.

**Aim:**

To identify and characterize biosignatures underlying various COVID-19 disease phases in an integrative multi-omics data analysis.

**Method:**

We leveraged a multi-omics network-based approach to integrate transcriptomics, metabolomics, proteomics, and lipidomics data. The World Health Organization Ordinal Scale WHO Ordinal Scale was used as a disease severity reference to harmonize COVID-19 patient metadata across two studies with independent data. A unified COVID-19 knowledge graph was constructed by assembling a disease-specific interactome from the literature and databases. Disease-state specific omics-graphs were constructed by integrating multi-omics data with the unified COVID-19 knowledge graph. We expanded on the network layers of multiXrank, a random walk with restart on multilayer network algorithm, to explore disease state omics-specific graphs and perform enrichment analysis.

**Results:**

Network analysis revealed the biosignatures involved in inducing chemokines and inflammatory responses as hubs in the severe and moderate disease phases. We observed distinct biosignatures between severe and moderate disease phases as compared to mild-moderate and mild-severe disease phases. Mild COVID-19 cases were characterized by a unique biosignature comprising C-C Motif Chemokine Ligand 4 (*CCL4*), and Interferon Regulatory Factor 1 (*IRF1*). Hepatocyte Growth Factor (HGF), Matrix Metallopeptidase 12 (*MMP12*), Interleukin 10 (*IL10*), Nuclear Factor Kappa B Subunit 1 (*NFKB1*), and suberoylcarnitine form hubs in the omics network that characterizes the moderate disease state. The severe cases were marked by biosignatures such as Signal Transducer and Activator of Transcription 1 (*STAT1*), Superoxide Dismutase 2 (*SOD2*), *HGF,* taurine, lysophosphatidylcholine, diacylglycerol, triglycerides, and sphingomyelin that characterize the disease state.

**Conclusion:**

This study identified both biosignatures of different omics types enriched in disease-related pathways and their associated interactions (such as protein-protein, protein-transcript, protein-metabolite, transcript-metabolite, and lipid-lipid interactions) that are unique to mild, moderate, and severe COVID-19 disease states. These biosignatures include molecular features that underlie the observed clinical heterogeneity of COVID-19 and emphasize the need for disease-phase-specific treatment strategies. The approach implemented here can be used to find associations between transcripts, proteins, lipids, and metabolites in other diseases.

## Background

Coronavirus Disease-2019 (COVID-19) is a contagious respiratory disorder caused by Severe Acute Respiratory Syndrome Coronavirus 2 (SARS-CoV-2), a newly emerged β coronavirus belonging to the Coronaviridae family (Karim and Khan, 2020). Since its discovery in Wuhan, China, in December 2019, COVID-19 established itself as a devastating global pandemic that has created disruptions across healthcare, economic, and social systems (Chen et al., 2020).

COVID-19 is characterized by a range of clinical phenotypes that reflect the spectrum of disease severity (i.e., mild, moderate, and severe herein defined as disease phases). Disease phenotypes are broadly classifiable as asymptomatic and symptomatic with approximately 85% of infected patients (vaccinated and non-vaccinated) showing mild to moderate symptoms and approximately 15% of infected patients suffering from potentially life-threatening complications (Singhal, 2020). The mild to moderate disease phase includes disease conditions with few or no infection symptoms, hospitalization with either no oxygen therapy required or with oxygen given by mask or nasal prongs, and no fatalities. In contrast, the severe disease phase includes disease conditions that, besides death, could involve one or a combination of hospitalization, oxygen therapy involving mechanical ventilation, respiratory failure, and significant immune dysregulation (Karim and Khan, 2020).

Each COVID-19 disease phase (i.e., mild, moderate, and severe) is marked by changes in omics profiles with corresponding changes in the expression levels of biosignatures (Blanco-Melo et al., 2020; Messner et al., 2020; Sun et al., 2020). In the context of this research, we define biosignatures as omics features that include proteins, transcripts, lipids, and metabolites. Although some of these biosignatures have a connection to the pathology of COVID-19 illness, not all of them actively impact the expressed disease phenotype when they are dysregulated. Different major dysregulated biosignatures including but not limited to Interferon Alpha 1 (*IFNA1*), Interferon Alpha Inducible Protein 6 (*IFI6*), Toll-Like Receptor 4 (*TLR4*) and interleukin-6 (*IL6*) are linked to host responses to COVID-19 (Bernardes et al., 2020; Ovsyannikova et al., 2020; Schulte-Schrepping et al., 2020; Aschenbrenner et al., 2021). Such biosignatures may serve not only as potential biomarkers to stratify patients according to disease severity and/or provide detailed prognostic information but could also contribute to the development of treatments that are more specifically targeted at particular disease states.

Several individual-omics (Fraser et al., 2020; Alqutami et al., 2021; Daamen et al., 2021; Jain et al., 2021; Patel et al., 2021; Zhong et al., 2021; Ciccarelli et al., 2022; Jia et al., 2022; Páez-Franco et al., 2022; Roberts et al., 2022; Suhre et al., 2022) and multi-omics COVID-19 investigations (Barh et al., 2020; Bernardes et al., 2020; Overmyer et al., 2020; Su et al., 2020; Stephenson et al., 2021; Sun et al., 2021; Suvarna et al., 2021; Chattopadhyay et al., 2022; Gygi et al., 2023) have identified biosignatures that are associated with disease progression. Individual omics studies provide specific insights into the contributions/manifestations of biosignatures at that omics level during disease progression but have not accounted for the impact(s) of other omics layers. Multi-omics studies present a means to collectively compare multiple omics data from different experiments either on the same samples or across studies, to yield a more holistic understanding of the biochemical underpinnings of COVID-19 outcomes. However, few of these omics studies (Barh et al., 2020; Bernardes et al., 2020; Overmyer et al., 2020; Su et al., 2020; Stephenson et al., 2021; Sun et al., 2021; Suvarna et al., 2021; Chattopadhyay et al., 2022) focusing on identifying the biochemical drivers of COVID-19 clinical heterogeneity have computationally integrated multi-omics data from different study samples with existing biological knowledgebases to explore biosignatures of different omics types and their connections across different disease phases. We suggest that this kind of multi-omics data integration is essential when attempting to explain the molecular dynamics underpinning the heterogeneity of COVID-19 infections while accounting for both prior knowledgebase and data from independent studies.

Network-based integrative approaches have revolutionized multi-omics analyses by providing the framework to build on existing knowledgebases when using new data to infer interactions between multiple different omics profiles within the context of a graph representation (Agamah et al., 2022). This has been shown to provide the opportunity not only to elucidate interactions that can occur among all classes of biomolecules in a biological system but also, to prioritize biosignatures that could discriminate disease severity. The approach represents the biomolecules that are most indicative of differences between disease states (i.e., the biosignatures) as nodes in the graph and infers relationships between them. For this reason, we hypothesized that i) investigating biosignatures across different phases of COVID-19 disease will provide insights into the molecular underpinnings of the enormous clinical heterogeneity of COVID-19 and ii) associations between biosignatures within a biologically meaningful network would permit the prioritization of biosignatures that discriminate between the disease states and could yield leads for potential drug targets.

Our study implemented a multi-omics network-based approach to identify and characterize biosignatures underlying various COVID-19 disease phases by integrating transcriptomics, metabolomics, proteomics, and lipidomics data (each representing a different data layer) with known interactome data (i.e., our present knowledgebase). We built disease-state specific omics-graphs and applied a network diffusion-based method to predict biosignatures and their associated interactions—both within and between omics layers—that are linked to the various COVID-19 disease phases. This is a form of intermediate-stage multi-omics integration or a multi-dimensional integration approach where multi-omics data are integrated simultaneously during the analysis (Adossa et al., 2021; Agamah et al., 2022).

The major contributions of this work are; 1) a new method of harmonizing patient disease severity metrics by leveraging the WHO Ordinal Scale (WOS) and patient metadata; 2) the assembly of a unified COVID-19 knowledge graph from different curated sources of interactome data (our resent knowledgebase); 3) the construction of a disease-state specific omics-graph by integrating curated transcriptomics, proteomics, lipidomics, and metabolomics datasets from two different multi-omics studies; and 4) identified biosignatures and their associated interactions that are shared and/or unique to mild, moderate, and severe COVID-19 disease-states. Overall, this study identifies biosignatures that discriminate between disease states. These results suggest that COVID-19 disease severity is influenced by interactions across different omics layers. Importantly, this study’s reproducible analysis pipelines offer a valuable tool for identifying biosignatures and interactions not just in COVID-19, but also in other diseases with distinct stages.

## Materials and Methods

### Study design and procedures

**FIGURE 1:**
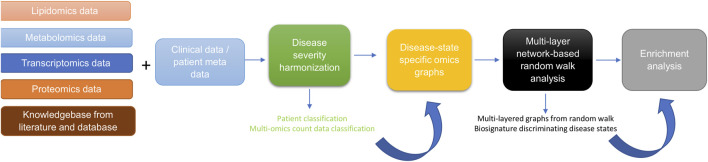
Diagram illustrating the workflow implemented in this study. The workflow begins with curating lipidomics, metabolomics, transcriptomics, proteomics data, and their associated patient metadata and knowledge graph from literature and databases. Next, we leveraged the patient metadata to perform disease severity harmonization. To harmonize the clinical severity of patients, we used the WOS as the reference for classifying disease severity into three disease states, such that: 1) mild disease state represents COVID-19 patients with WOS 1–2, 2) moderate disease state represents COVID-19 patients with WOS 3–4, and 3) severe disease state represents COVID-19 patients with WOS 5–9. We then used the harmonized information to split the omics datasets according to disease severity before constructing coexpression networks and disease-state specific omics-graphs. We then performed random walk analysis on the graphs to predict biosignatures discriminating the various disease states. Finally, we performed enrichment analysis on the proteins, transcripts, metabolites, and lipids.

The approach for this study (Figure 1) consists of five main steps including: 1) data curation and pre-processing; 2) disease severity harmonization; 3) construction of disease-state specific omics-graphs; 4) multi-layer network-based random walk analysis; and 5) enrichment analysis.

## Data sources

### Multi-omics experimental data

Multi-omics experimental datasets from two independent studies were used in this study; quantified blood plasma transcript, protein, and metabolite count data from (Su et al., 2020), and transcript, protein, metabolite, and lipid count data from (Overmyer et al., 2020). For both studies by (Su et al., 2020), and (Overmyer et al., 2020), patient recruitment and sample collection occurred in 2020 during the peak of COVID-19 cases. The methodologies utilized for data collection and patient treatment in both studies are different and are outlined in the original studies. For (Su et al., 2020), the study samples consisted of 139 COVID-19 patients (60 males and 79 females) and 258 healthy controls (Supplementary Data Table S1). These enrolled COVID-19 patients had an age range from 18 to 89 years (median = 58). The patients from which blood is drawn were classified as WHO Ordinal Scale (WOS) = 3–4 (*n* = 83) and WOS = 5–7 (*n* = 47). The authors used an unpaired Wilcoxon test to determine the statistical difference between WOS = 3–4 and WOS = 5–7, and *p* values were FDR adjusted. The Overmyer et al., 2020), study samples were collected from 128 adult patients of which 102 (64 males and 38 females) were COVID-19-positive and 26 (13 males and 13 females) were negative (Supplementary Data Table S1). There were no significant differences between the average ages of males and females in either the COVID-19 positive group—(61.3 years for females and 63.1 years for males; (*p*-value = 0.56; calculated using *t*-test) or the COVID-19 negative group (59.5 years for females and 67.0 years for males; *p*-value = 0.25).

### Protein-protein interactome

We retrieved 1832 names of human genes associated with COVID-19 from the DisGeNET database version 5 (Piñero et al., 2020). GeneMANIA (Franz et al., 2018) was used to generate an interactome for 1,692 of these genes (140 were unrecognized by the GeneMANIA database). Interactions between genes were based on co-expression, physical interaction, co-localization, shared protein domains, genetic interaction, or predictions from manual curation.

### Metabolite-metabolite interactome

A co-expression network was constructed from the metabolomics measurement data. Specifically, Pearson’s correlations were used to estimate the individual relationships between features for every pre-processed omics measurement using an in-house R script Highly correlated pairwise interaction scores ≥0.7 were used to select components of the metabolite-metabolite interactome that was used for downstream analysis. The threshold of 0.7 was determined after carefully evaluating the structural behaviour of the co-expression network and the observed associations at different thresholds.

### Lipid-lipid interactome

A co-expression network was constructed from the lipidomics measurement data. Similar to the process implemented for metabolite-metabolite interactome above, highly correlated pairwise interaction scores ≥0.7 were used to select components of the lipid-lipid interactome that was used for downstream analysis.

### COVID-19 knowledge graph

We further included a multi-modal, knowledge model of COVID-19 pathophysiology (COVID-19 Knowledge Graph version 0.0.2, https://github.com/covid19kg/covid19kg) (Domingo-Fernández et al., 2021). The graph incorporates nodes, covering 10 entity types (e.g., proteins, genes, chemicals, and biological processes) and relationships between the nodes, and we considered only protein, genes, transcripts, lipids, and metabolites node types and their interactions for downstream analysis.

### Cross-layer interactome

We retrieved protein-transcript, metabolite-protein, and lipid-protein associations from (Su et al., 2020), and (Overmyer et al., 2020), and used these to construct a bipartite graph for network analysis.

### Harmonizing the clinical severity of patients

Patient sample metadata from both the (Su et al., 2020), and (Overmyer et al., 2020), sources were used for disease severity harmonization. For (Su et al., 2020), metadata, the disease state linked to the samples was described using the WHO Ordinal Scale (WOS) based on specific categories and characteristics including 1) uninfected—no evidence of infection; 2) infected but ambulatory with no limitation of activities; 3) infected with limitation of activities but still ambulatory; 4) hospitalized with no or mild oxygen therapy; 5) hospitalized with oxygen administered by mask or nasal prongs; 6) hospitalized with non-invasive ventilation or high-flow oxygen; 7) hospitalized with intubation and mechanical ventilation; 8) hospitalized, with intubation and mechanical ventilation together with additional organ support; and 9) death.

In the metadata (Overmyer et al., 2020), disease severity was quantified using hospital-free days at day 45 (HFD-45) scores: a composite outcome variable that accounts for the length of hospital stay. The utility of the HFD-45 score is derived from the fact that severe COVID-19 patients are those who are admitted to the hospital the longest as they require ventilatory support, while those with the most extreme cases die during hospitalization (Overmyer et al., 2020). The variable assigns a zero value (0-free days) to patients with severe disease who remain admitted longer than 45 days or die due to respiratory deterioration while admitted, and higher values of HFD-45 to patients with shorter hospitalizations and milder disease severity.

To harmonize the clinical severity of patients, we used the WOS as the reference for classifying disease severity into three disease states, such that: 1) mild disease state represents COVID-19 patients with WOS 1–2, 2) moderate disease state represents COVID-19 patients with WOS 3–4, and 3) severe disease state represents COVID-19 patients with WOS 5–9.

The (Overmyer et al., 2020), sample metadata included the following variables: 1) ICU Status (an indicator variable of the patient’s ICU status), 2) HFD-45, 3) Acute Physiologic Assessment and Chronic Health Evaluation II (APACHE II) Score (an indicator variable ranging from 0 (best health) to 71 (worst health) based on physiologic variables, age, and health conditions), and 4) Mechanical Ventilation Status (MVS) (an indicator variable describing the patient’s mechanical ventilation status). There was a correlation between the HFD-45 and ICU status with APACHE II and MVS. These variables could feasibly be mapped onto the WOS scale, knowing that the WOS scale is primarily based on respiratory status and oxygen/ventilation support. Accordingly, we leverage this metadata to map characteristics of the (Overmyer et al., 2020) study patients on the WOS. Specifically, we assigned: a mild disease state to COVID-19 patients with HFD-45 between 29–45 with no time spent in the ICU, a moderate disease state to COVID-19 patients with HFD-45 between 29–45 who spent time in the ICU, or an HFD-45 between 21–28 regardless of time spent in the ICU, and severe disease state to COVID-19 patients with HFD-45 less than 20 regardless of time spent in the ICU.

### Data pre-processing

We conducted a two-step data pre-processing operation on the omics experimental data using a custom script. Outlier and missing values were removed, and data were normalized. Samples with more than 20% missing data in a certain data type were excluded. Similarly, biological features such as mRNA expression, with more than 20% of values missing across patients were dropped from the data. Z-score normalization was then applied such that each feature of the data (samples as columns and features as rows) had an average of 0 and a standard deviation of 1.

### Feature mapping to unified identifiers

To ensure that the feature labels were unified, transcript and protein identifiers were mapped to gene-level IDs using the UniProt (https://www.uniprot.org/) and NCBI databases (https://www.ncbi.nlm.nih.gov/gene/). Metabolite and lipid name descriptors were maintained for all analyses other than functional analysis for which KEGG or PubChem IDs were used.

### Building a unified knowledge graph

We assembled a unified knowledge graph by merging the protein-protein interactome, the metabolite-metabolite interactome, the lipid-lipid interactome, and the extracted data from the COVID-19 knowledge graph using a custom script (Domingo-Fernández et al., 2021).

### Building disease-state specific omics-graphs

Protein-protein, transcript-transcript, lipid-lipid, and metabolite-metabolite co-expression networks for the various COVID-19 disease states (i.e., mild, moderate, severe) were constructed based on an integrated unified knowledge graph, and the pre-processed omics data using an R script. From the (Su et al., 2020), omics data, we constructed three co-expression networks (protein-protein, transcript-transcript, and metabolite-metabolite) for each disease state. Likewise, we constructed four co-expression networks (protein-protein, transcript-transcript, lipid-lipid, and metabolite-metabolite) for each disease state from the (Overmyer et al., 2020), data. This was achieved by evaluating the correlation between the expression of each linked feature pair where each feature is represented in the unified knowledge graph (i.e., transcript-transcript, protein-protein, metabolite-metabolite, lipid-lipid feature pairs). Pearson’s correlations were used to estimate the individual relationships between features for every pre-processed omics measurement using an in-house R script.

The interaction between feature pairs is represented by the Pearson Correlation Coefficient (PCC) ranging between −1 (perfect negative correlation) and 1 (perfect positive correlation), with values of zero representing no correlation. We further rescaled the PCC score for each pairwise interaction by computing the absolute value to attain positive scores ranging between 0 and 1. Additionally, we defined a threshold of 0.1 for edge filtering after careful consideration of the results at different threshold points. We suggest that these steps contributed significantly to addressing the potential for spurious findings and contributed significantly to the reliability of our findings. The co-expression networks constructed from each single omics dataset formed the baseline for the network integration. Specifically, the co-expression networks of the same omics data type constructed from the two independent studies were integrated by merging the networks to construct four omics-specific graphs (one for each omics type) for each of the three disease states.

## Random walk network analysis

### COVID-19 disease state graph exploration by a random walk with restart

We adapted multiXrank (Baptista et al., 2022; Baptista et al., 2024), a random walk with restart on a multilayer network algorithm to explore the disease-state specific omics-graphs. This algorithm was chosen because it enables random walk with restart on any kind of multilayer network generated from different data sources as compared to other methods that are limited in the combination and heterogeneity of networks that they can handle (Agamah et al., 2022). We modified the configuration script for the algorithm to accept four disease-state specific omics-graphs for our analysis.

For network exploration on each disease state, the disease-state specific omics-graphs, cross-layer interactome, and seed nodes were used as inputs for the algorithm. Outputs were multi-layered graphs that described the exploration of the seed nodes across the different disease-state specific omics-graphs and a list of features in each disease-state specific omics-graph ranked according to their proximity to seed nodes.

The parameter values for global restart probability (set to 0.7), and inter-layer jump probability in a given disease-state specific omics-graph (set to 0.5), were maintained. The probability to restart in a specific layer of a specific disease-state specific omics-graph was set to 1: a setting that meant the disease-state specific omics-graph was classifiable as a monoplex network.

The probability of restarting in a specific disease-state specific omics-graph was set to 0. This meant that the random walker stayed within the network within which it began with a probability equal to 1.

To achieve homogeneous exploration, the initial probability of jumping across different disease-state specific omics-graphs was set to 0.25 in consideration of the four disease-state network layers.

Briefly, the first step of the algorithm is to create adjacency matrices for the input graphs, followed by computing different transition probabilities of the random walk with restart on the graphs. The probabilities are estimated based on the concept that an imaginary particle starts a random walk from the seed node to other nodes in the network. These different transition probabilities describe the walks within a graph and the jumps between graphs. A higher probability score (close to 1) suggests a higher likelihood of walking or jumping between graphs.

### Identifying seed nodes for multi-layered network exploration

To select seed nodes for the analysis, we implemented two approaches; 1) a data-driven approach where we selected, after merging the different co-expression networks, the features with the highest node integrated centrality score in each omics layer as seeds, and 2) a hypothesis-driven approach where we selected seeds based on their impact on disease severity to test the hypothesis of their differential associations with mild, moderate, and severe COVID-19 disease states. Hypothesis-driven has the advantage of bringing the question being investigated into focus by designing the model with a specific biological hypothesis in mind and exploring variations across disease phases while the data-driven, enables a more unbiased and informed model through computationally intensive use of the data (Arazi et al., 2013; Eriksson et al., 2022). Although hypothesis- and data-driven modeling approaches are not mutually exclusive, it is worth noting that this diversity is beneficial: most model-building tools and models have a specific and clear role, however at the same time, combining hypothesis- and data-driven approaches in an interoperable way, provide an immense impact on our understanding of the disease phases as modelling and integrating data at different biological scales (Arazi et al., 2013; Eriksson et al., 2022).

For the data-driven approach, the features were ranked by leveraging the node degree, closeness, betweenness, and eigenvector centrality metrics to compute an integrated score (see Supplementary Data Equations S1–S3). These centrality metrics provide insight into the importance of a node. For instance, the closeness metric measures how close a node is to all other nodes in the network. A lower closeness centrality score indicates the node is on average closer to other nodes, potentially making it a faster “information hub.” The degree metric measures the number of edges (connections) a node has with other nodes. A higher degree means the node has more direct connections, suggesting it might be more influential or receive more information flow. The betweenness metric captures how often a node lies on the shortest path between other pairs of nodes. A higher betweenness centrality score suggests the node acts as a crucial bridge for information flow within the network. The eigenvector metric considers not just the number of connections a node has, but also the “importance” of its neighbours. Nodes with high eigenvector centrality are considered influential due to their connections to other influential nodes.

### Ranking candidate multi-omics features for COVID-19 disease states

All the network nodes were scored and ranked by the algorithm according to their proximity to the seed nodes (Baptista et al., 2022). The computed score was the geometric mean of the node’s proximity to the seeds.

### Enrichment analysis

#### Metabolite pathway

Metabolite pathway enrichment analysis was performed using the MetaboAnalyst 5.0 online Pathway Analysis tool (Pang et al., 2021) (accessed 26 January 2023). Metabolite names were entered as KEGG IDs, and when necessary, metabolite names were automatically adjusted to match the nomenclature recognized by MetaboAnalyst (e.g., *Hydroxypropanoate* as *hydroxypropanoic acid*). Using high-quality KEGG metabolic pathways as the backend knowledgebase, we used the hypergeometric statistical test to examine the overrepresentation of metabolites predefined in the KEGG pathway present in the queried metabolites. This approach determined whether a particular group of compounds was represented more than expected by chance within the user-uploaded compound list.

#### Lipid pathway

We conducted lipid pathway enrichment analysis using the LIPEA online Pathway Analysis tool (Acevedo et al., 2018) (accessed 26 January 2023). Lipid names were entered as a compound list and, when necessary, these names were adjusted to match the nomenclature recognized by LIPEA. The resource computes an unadjusted *p*-value on overrepresented pathways, and a *p*-value adjusted with Bonferroni correction to reduce false positives.

#### Gene ontology analysis

Protein/transcript enrichment analysis was performed using the online Enrichr Gene Ontology Resource (Kuleshov et al., 2016) (accessed 26 January 2023). The resource provides gene-set libraries made of a set of related genes that are associated with a functional concept such as a biological pathway or process (Kuleshov et al., 2016). Gene ID identifiers were used as input for the enrichment analyses. The resource computes Fisher’s exact test followed by a correction based on a mean rank and standard deviation from the expected rank for each term in each gene-set library (Kuleshov et al., 2016).

## Results

### Harmonized clinical severity between patients’ metadata

**FIGURE 2:**
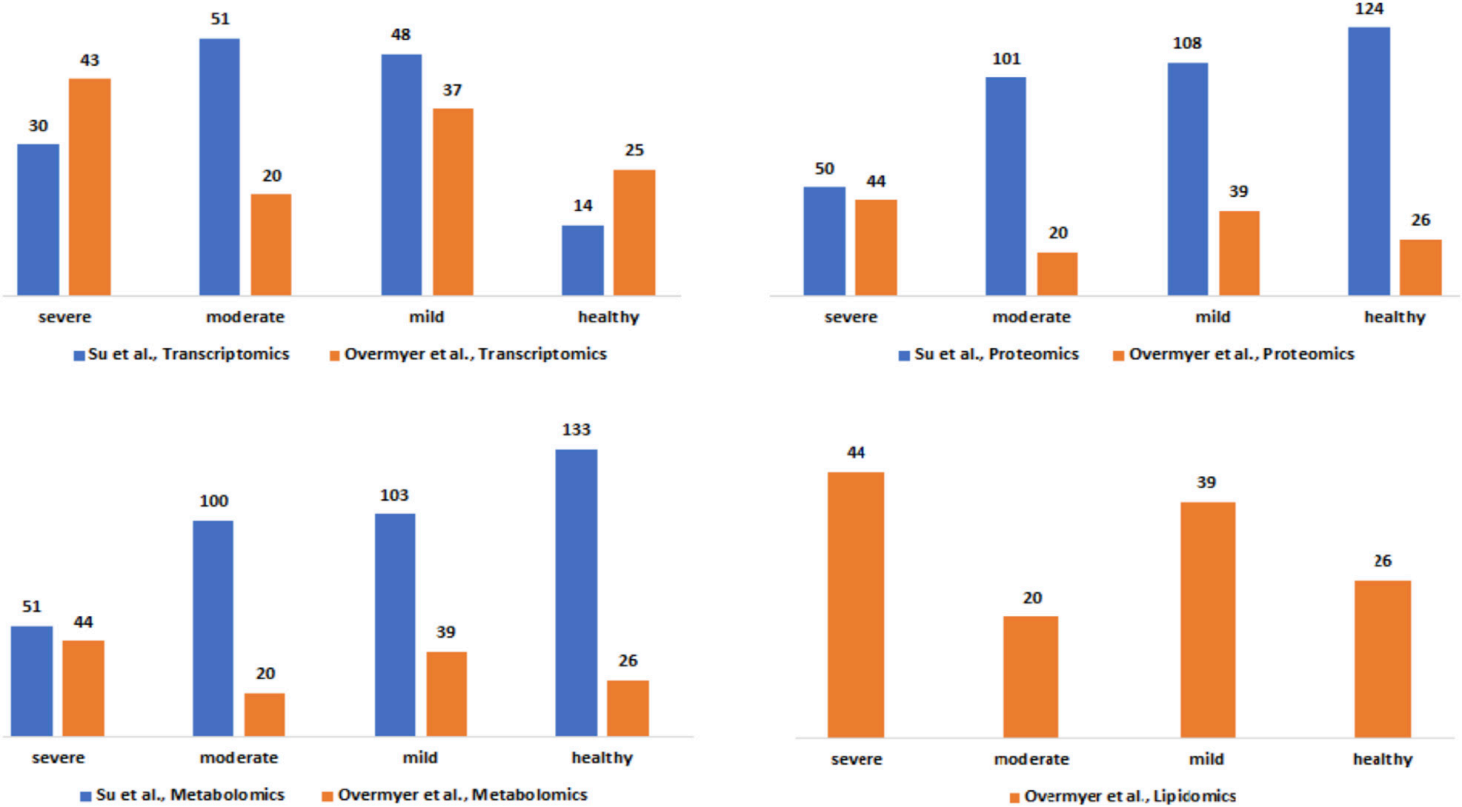
Description of the Su et al., and Overmyer et al., samples based on the omics data type and the disease severity levels after the harmonization process.

The clinical severity harmonization was a crucial step in integrating and analysing data from multiple sources and platforms to gain a more comprehensive understanding of biological systems and disease mechanisms. There were more severe samples than any other in the (Overmyer et al., 2020) dataset followed by mild and then moderate samples (Figure 2). For the (Su et al., 2020), dataset, there were similar numbers of mild and moderate samples and fewer severe samples. The classified samples (Supplementary Data Table S2) were used to split the omics counts data into disease states before constructing the disease-state specific omics-graphs.

### Integrative network-based multi-omics analysis

#### Construction of disease-state specific omics-graphs

**FIGURE 3:**
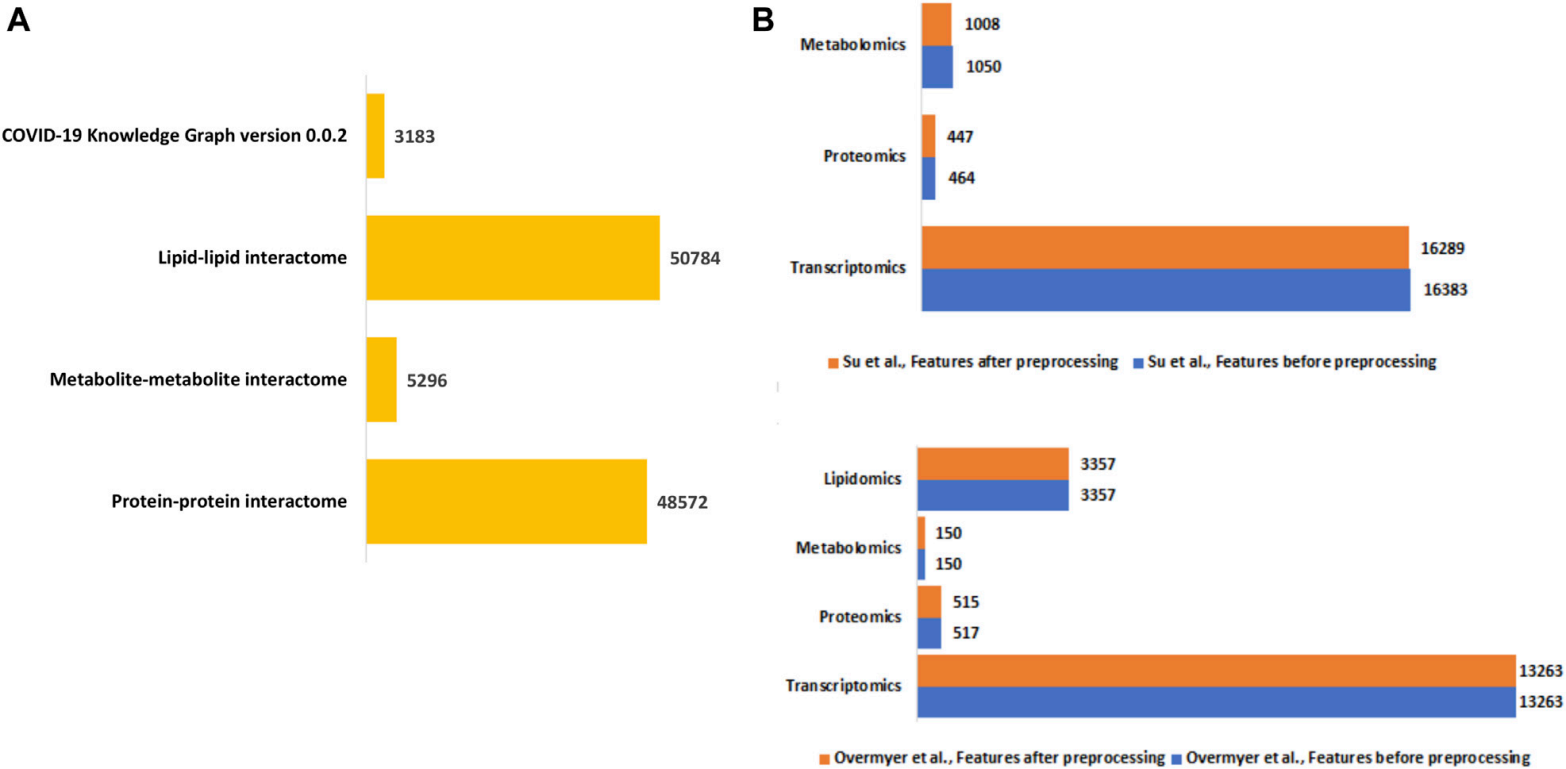
**(A)** Summary of the edge count in the interactome datasets used to construct the unified knowledge graph. **(B)** Distribution of features in the omics experimental datasets before and after data processing.

We constructed a unified knowledge graph (Figure 3A) comprising four different edge types by merging the protein-protein interaction data from GeneMANIA, metabolite-metabolite interactome, lipid-lipid interactome, and the extracted data from the COVID-19 knowledge graph. Importantly, the unified knowledge graph formed the basis for integrating the processed multi-omics data (Figure 3B) and constructing disease-state specific omics-graphs for mild, moderate, and severe COVID-19 disease states (Supplementary File S1). Additionally, the disease-state specific omics-graphs enabled us to investigate COVID-19 disease states in the context of specific omics data types.

#### Identified seed nodes for network exploration

The random walk method is a technique for detecting the flow of biological information throughout networks. The concept behind the random walk method is such that a hypothetical particle exploring the network structure takes random discrete steps (walks) in some direction from a seed node (Agamah et al., 2022). The walk explores different layers based on the premise that nodes related lie close to each other in the network (Valdeolivas et al., 2019).

We selected the seed nodes at which random walks began (Table 1) using both data-driven and hypothesis-driven approaches. Whereas for the data-driven approach, we selected a seed primarily based on network topology by computing an integrated node centrality metric score (Supplementary Data), for the hypothesis-driven approach, we selected nodes based on previously reported associations of molecular features with COVID-19 disease states and to test the differential association these nodes with disease outcomes.

**TABLE 1:** Selected seeds for random walk network exploration.

Approach	Seed node	Integrated centrality score	Feature type
Data-driven	STAT1	53,529.0403	Transcript
	SOD2	2,215.5746	Protein
	3-hydroxyoctanoate	1,506.9998	Metabolite
	Unknown_mz_815.61548_+_RT_27.063	9936.9781	Lipid
Hypothesis-driven	IL6R	252.0804	Transcript
	IL6	2,208.0288	Protein

For the data-driven approach, one selected seed node was the Signal Transducer And Activator Of Transcription 1 (*STAT1*) from the transcriptomics layer. *STAT1* is known to be involved in immune responses and antiviral activity (Lucas et al., 2020) and is reported to be upregulated in mild and severe COVID-19 cases, with the phosphorylation of the gene highly enhanced in severe disease states (Rincon-Arevalo et al., 2022). Another selected seed node from the proteomics layer was Superoxide Dismutase 2 (*SOD2*), an essential antioxidant enzyme that protects cells from superoxide radical anions which are known to be significantly under-expressed in the plasma (Žarković et al., 2022), and in the lung cells of severely infected COVID-19 patients (Zarkovic et al., 2022). From the metabolomics layer, we used 3-hydroxyoctanoate, as a seed: 3-hydroxyoctanoate is a metabolite of medium-chain fatty acid oxidation that has been identified as a marker for primary defects of beta-hydroxy fatty acid metabolism and it is a conjugate acid to 3-hydroxyoctanoate, a biomarker of asymptomatic COVID-19 infection that is involved in important pathways such as the activation of macrophage and platelet aggregation (Franz et al., 2018). From the lipidomics layer, we identified unknown_mz_815.61548_+_RT_27.063, an uncharacterized lipid as a seed.

For the hypothesis-driven approach, we selected interleukin-6 (*IL6*) and interleukin-6 receptor (*IL6R*) as seeds. Besides the pathologic roles of these molecules in immune-inflammatory diseases such as COVID-19, it has been hypothesized that inhibition of *IL6* receptors (*IL6Rs*) by tocilizumab ameliorates the symptoms of severe COVID-19 and reduces mortality (Kaye and Siegel, 2020; Samaee et al., 2020). We aimed to find out how *IL6* and *IL6R* influence disease severity.

#### Random walk analysis on disease-state specific omics-graphs using data-driven seeds

We used various omics features (Table 1) as seed nodes for the random walk analysis. Subsequently, features in each disease-state specific omics-graph were ranked by their proximity to the seeds. The generated multi-layered graphs (accessible at http://cytoscape.h3africa.org), describing the exploration of the seeds during the random walk analysis for each disease state, suggested that cross-layer interactions between the different omics data types influence disease severity. This is evidenced particularly in the protein-metabolite (e.g., *HGF* and 1-palmityl-GPC, *HGF* and 6-bromotryptophan), transcript-metabolite (e.g., *CCL2* and taurine, *CCL2* and 1-(1-enyl-palmitoyl)-2-oleoyl-GPC)), and protein-transcript (e.g., *HGF* and *HLA-B*) interactions. For each omics layer, we identified highly connected features (Table 2) forming large subnetworks and defined these as “key hubs.”

**TABLE 2:** Key hubs identified in the disease-state specific omics-graphs upon using seeds from the data-driven approach.

Disease state	Omics layer	Feature/Hub	Proximity to seeds score
Mild	Transcriptomics	Coagulation Factor XI (*F11*)	0.00555
		C-C Motif Chemokine Ligand 4 (*CCL4*)	0.00548
	Proteomics	Interferon Regulatory Factor 1 (*IRF1*)	0.00115
	Metabolomics	3-hydroxydecanoate	0.02761
		3-hydroxyhexanoate	0.02736
	Lipidomics	Unknown_mz_834.66107_+_RT_26.843	0.00177
		Unknown_mz_838.69214_+_RT_27.059	0.00175
Moderate	Transcriptomics	Hepatocyte Growth Factor (*HGF*)	0.00542
		Matrix Metallopeptidase 12 (*MMP12*)	0.00528
	Proteomics	Interferon Regulatory Factor 1 (*IRF1*)	0.00114
	Metabolomics	3-hydroxydecanoate	0.02761
		3-hydroxyhexanoate	0.02733
	Lipidomics	Unknown_mz_834.66107_+_RT_26.843	0.00177
		Unknown_mz_553.38593_+_RT_19.676	0.00167
Severe	Transcriptomics	Hepatocyte Growth Factor (*HGF*)	0.00589
		Matrix Metallopeptidase 12 (*MMP12*)	0.00568
	Proteomics	Interferon Regulatory Factor 1 (*IRF1*)	0.00118
	Metabolomics	3-hydroxydecanoate	0.02761
		3-hydroxyhexanoate	0.02733
	Lipidomics	Unknown_mz_834.66107_+_RT_26.843	0.00177
		Unknown_mz_553.38593_+_RT_19.676	0.00167
		Unknown_mz_736.64563_+_RT_24.633	0.00167

#### Random walk analysis on disease-state specific omics-graphs using hypothesis-driven seeds

Like the network exploration using data-driven seeds, we repeated the analysis with hypothesis-driven seeds. We observed from the multi-layer graphs (accessible at http://cytoscape.h3africa.org) that *IL6* interaction with features of different omics data types increased with disease severity, thus indicating the differential association of *IL6* with the different disease states. On the other hand, we observed *IL6R* interactions mainly with proteins and transcripts to increase with disease severity as compared to interactions with metabolites. The results suggest *IL6* interaction with proteins (e.g., *IFNB*, *IFIT3*), transcripts (e.g., *CXCL1*, *CXCL2, CCL3*), and metabolites (e.g., 1-(1-enyl-palmitoyl)-GPC, 1-(1-enyl-palmitoyl)-2-oleoyl-GPC (P-16:0/18:1)) may contribute to its significant role in disease severity. Similar to analysis using data-driven seeds, we identified key hubs from each omics layer (Table 3). The hubs with zero proximity scores establish subnetworks with no direct interaction with seed nodes (*IL6* and *IL6R*) (as shown in http://cytoscape.h3africa.org).

**TABLE 3:** Key hubs identified in the disease-state specific omics-graphs upon using seeds from the hypothesis-driven approach.

Disease state	Omics layer	Feature	Proximity to seeds score
Mild	Transcriptomics	C-C Motif Chemokine Ligand 4 (*CCL4*)	0.00659
		C-C Motif Chemokine Ligand 2 (*CCL2*)	0.005629
	Proteomics	Interleukin-7 Receptor (*IL7R*)	0.00368
	Metabolomics	tricosanoyl sphingomyelin (d18:1/23:0)*	0.0
		suberate (C8-DC)	0.0
		stearoylcholine*	0.0
	Lipidomics	Unknown_mz_765.5752_-_RT_20.235	0.0
		Unknown_mz_765.3349_-_RT_6.822	0.0
		Unknown_mz_765.3349_+_RT_6.842	0.0
Moderate	Transcriptomics	Nuclear Factor Kappa B Subunit 1 (*NFKB1*)	0.00545
		Interleukin 10 (*IL10*)	0.00539
	Proteomics	Interleukin-7 Receptor (*IL7R*)	0.00339
	Metabolomics	sphingomyelin (d18:1/25:0, d19:0/24:1, d20:1/23:0, d19:1/24:0)*	0.0
		sulfate of piperine metabolite C16H19NO3 (2)*	0.0
		suberoylcarnitine (C8-DC)	0.0
		suberate (C8-DC)	0.0
	Lipidomics	Unknown_mz_768.54962_-_RT_23.304	0.0
		Unknown_mz_763.31421_+_RT_5.276	0.0
Severe	Transcriptomics	C-C Motif Chemokine Ligand 4 (*CCL4*)	0.00612
		C-X-C Motif Chemokine Ligand 1 (*CXCL1*)	0.00549
	Proteomics	Interleukin-7 Receptor (*IL7R*)	0.00358
	Metabolomics	sphingomyelin (d18:2/21:0, d16:2/23:0)*	0.0
		tartronate (hydroxymalonate)	0.0
		sulfate of piperine metabolite C18H21NO3 (1)*	0.0
	Lipidomics	Unknown_mz_765.60468_+_RT_16.383	0.0
		Unknown_mz_766.75885_+_RT_1.234	0.0
		Unknown_mz_766.69232_+_RT_28.305	0.0

Compounds for which a matching pure standard was not available for confirmation are denoted by adding an asterisk (*) symbol after the name of the metabolite.

Features with 0.0 proximity score are part of subnetworks that have no direct edge with the seed nodes.

#### Evaluating features and interactions of generated multi-layered graphs

In this section, we dissect different multi-layered graphs generated from random walk analysis to examine common and unique feature interactions related to disease severity. The multi-layered networks generated for each disease state contain four types of nodes (proteins, transcripts, lipids, and metabolites), and at most six types of edges (transcript-transcript, protein-protein, protein-transcript, protein-metabolite, metabolite-metabolite, lipid-lipid). We did not observe protein-lipid edge types across all the networks. This observation is because the seed exploration prioritizes nodes that have an either direct or indirect connection to the seeds (i.e., related to the seeds), thus no observed protein-lipid edge type is an indication that there was limited bipartite data that captures interactions between nodes and seeds of interest.

#### Evaluating multi-layered graphs generated using data-driven seeds

We evaluated the feature interactions (Supplementary File S2) present in the multi-layer graphs (accessible at http://cytoscape.h3africa.org) identifying 204 interactions associated with a specific disease state of which 79%, 15%, and 6% are transcript-transcript, lipid-lipid, and protein-protein interactions respectively. Of these interactions, most (88%) are associated with the mild disease state. Additional investigation of 263 interactions associated with only two of the three disease states revealed more pairwise interactions common between the moderate and severe disease states as compared to those in common between the mild-moderate and mild-severe disease state pairs (Supplementary File S2). Also, an investigation of 397 interactions common across the three disease states revealed a higher proportion of interactions between transcripts associated with various cellular processes and about 1% each for metabolite-metabolite, protein-protein, and protein-metabolite interactions (Supplementary File S2).

**FIGURE 4:**
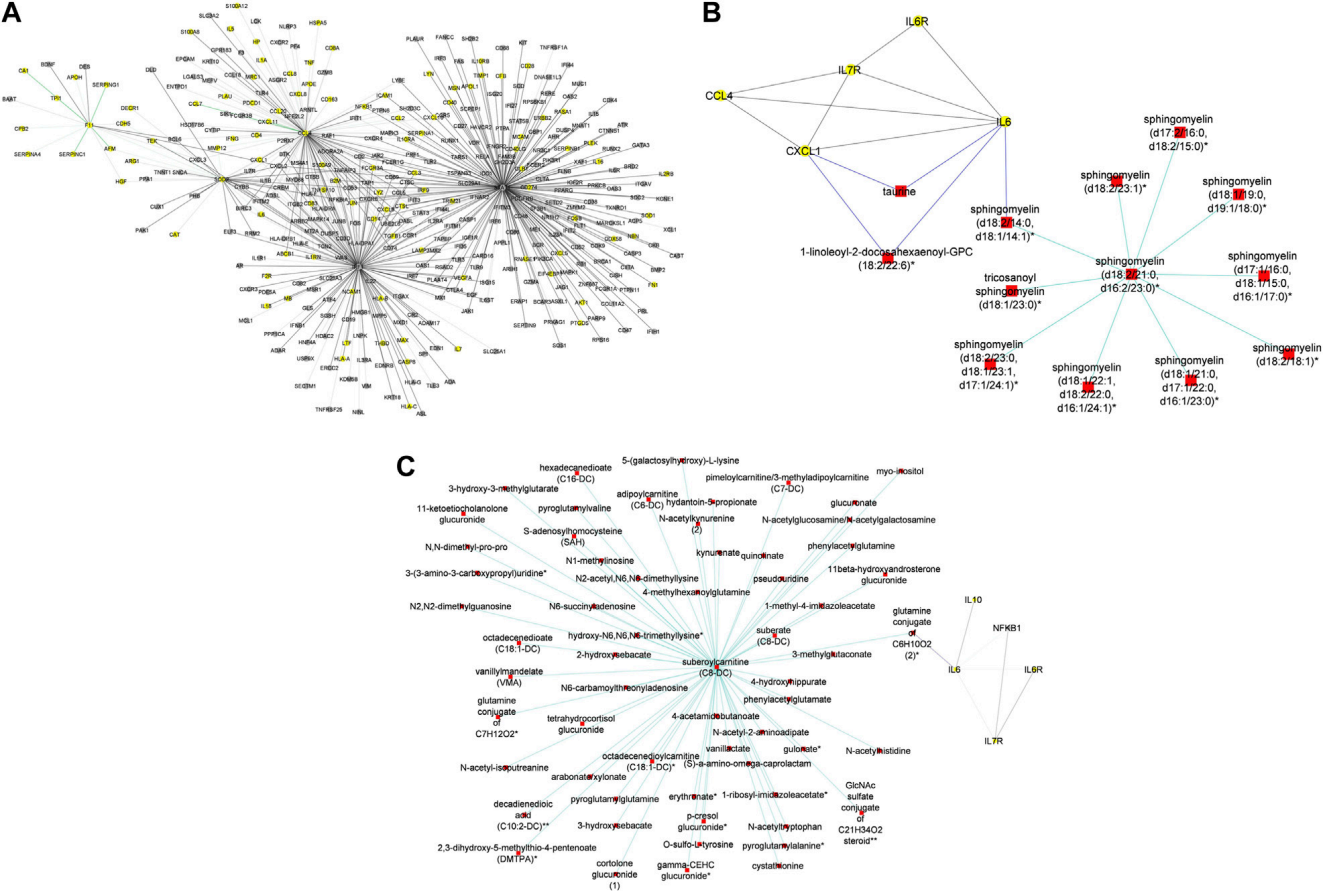
**(A)** Graph representation of subnetworks formed by hubs CCL4, F11, and IRF1, that establish direct interaction with seed nodes (STAT1 and SOD2) as observed in the multi-layered graph generated for the mild disease state. The graph highlights the interaction of these hubs with other molecular features including proteins (yellow nodes) and transcripts (grey nodes) **(B)** Graph representation of subnetworks formed by hubs HGF, IRF1, and MMP12, that establish direct interaction with seed nodes (STAT1 and SOD2) as observed in the multi-layered graph generated for the moderate disease state. The graph highlights the interaction of these hubs with other molecular features including proteins (yellow nodes), transcripts (grey nodes), and metabolites (red nodes). **(C)** Graph representation of subnetworks formed by hubs HGF, and IRF1 that establish direct interaction with seed nodes (STAT1 and SOD2) as observed in the multi-layered graph generated for the severe disease state. The graph highlights the interaction of these hubs with other molecular features including proteins (yellow nodes), transcripts (grey nodes), and metabolites (red nodes). The grey edges represent transcript-transcript interactions, the yellow edges represent protein-protein interactions, the cyan edges represent metabolite-metabolite interactions, the green edges represent protein-transcript interactions and the blue edges represent both protein-metabolite and transcript-metabolite interactions. Compounds for which a matching pure standard was not available for confirmation are denoted by adding an asterisk (*) symbol after the name of the compound.

In addition to subnetworks formed by the seed nodes, we observed *CCL4, F11,* and *IRF1* interact directly with seed nodes *SOD2* and *STAT1* and form subnetworks in the multi-layered graph generated for the mild disease state. *CCL4* established interactions with human leucocyte antigen, co-stimulatory molecules (e.g., *CD2, CD4, CD8, CD83, CD53*, *CD3D HLA-DPA1, HLA-DPB1, HLA-DRA*), and other molecules expressed in monocytes and macrophages (e.g., *CCL5, CCL7, CCL8*) to be highly predominant in the mild disease state (Figure 4A). This observation may suggest a more efficient immune response to the virus among patients with mild disease as compared to those with moderate and severe disease, thus leading in the latter group to decreased recruitment of immune cells to the site of infection (Lucas et al., 2020).

We identified intra-omics interactions (specifically transcript-transcript and protein-protein) shared between mild-severe and mild-moderate disease state pairs. Notable interactions were among *STAT1*, interferons (e.g., *IFNB1, ISG20*) and *SOD2*. *STAT1* is involved in regulating T-cell activation and differentiation responses, thus regulating the pathogenesis of COVID-19. Therefore, associations between *STAT1* and T-cell receptors (e.g., *CD38, CD40, CD48, CD68*) in the mild-severe disease state, may suggest a more efficient role in the immune response to viral infections during mild disease state and higher *STAT1* activation in severe disease state contributing to the cytokine storm and hyperinflammation that are characteristic of severe COVID-19 (Hadjadj et al., 2020). Further, interactions between *STAT1* and interferons highlight the critical role of *STAT1* in the regulation of interferon-stimulated genes, because interferons are key cytokines in the immune response to viral infections. Also, interactions between interferons and other genes (e.g., *TNFRSF25*, *TLR3, MAPK1*) involved in interferon-mediated pathways, highlight the likelihood of innate and adaptive immune stimulatory effects.

A distinctive factor for severe-moderate compared to mild-moderate and mild-severe disease state pairs was the cross-layer interactions between protein-metabolite and protein-transcript. Particularly, Hepatocyte Growth Factor (*HGF*) and Matrix Metallopeptidase 12 (*MMP12*) interactions with metabolites (e.g., 6-bromotryptophan, cortolone glucuronide, 1-palmityl-2-linoleoyl-GPC, 1-(1-enyl-stearoyl)-2-linoleoyl-GPC, sphingomyelin) and proteins implicated in various cellular processes were highly predominant in the moderate disease state (Figure 4B). On the other hand, *HGF* cross-layer interaction in the severe disease state was predominant and not *MMP12* (Figure 4C) as observed in the moderate disease state.

*HGF* plays a critical role in tissue repair and regeneration, particularly in the liver and lungs, and has been found to have potential therapeutic effects in COVID-19 due to its ability to reduce lung injury and improve pulmonary function. Studies have shown that COVID-19 patients with severe respiratory symptoms have significantly lower levels of *HGF* in their blood and that treatment with *HGF* can reduce lung inflammation and prevent the progression of COVID-19-related lung injury (Xiao et al., 2020). *HGF* has also been shown to have antiviral effects against the SARS-CoV-2 virus *in vitro*, suggesting that it may help to inhibit viral replication in infected cells (Quartuccio et al., 2021; Wang et al., 2021). Thus, the observed interactions may suggest a protective role for *HGF* in ameliorating the progression of COVID-19, as well as a target for drug research (Quartuccio et al., 2021; Wang et al., 2021).

Evidence suggests that both *HGF* and *MMP12* levels are significantly elevated in the lungs of patients with severe disease (Perreau et al., 2021; Salomão et al., 2023). Particularly, elevated MMP12 levels play a role in controlling disease pathogenesis and lung injury, acknowledging that excessively elevated levels can disrupt the balance of the extracellular matrix, resulting in tissue damage (Salomão et al., 2023).

Therefore, the observed cross-layer interactions for *HGF* and *MMP12* in moderate and severe diseases suggest that cross-layer interactions influence clinical heterogeneity, thus influencing the dynamics of disease severity.

#### Evaluating multi-layered graphs generated using hypothesis-driven seeds

In this section, we evaluated the feature interactions (Supplementary File S2) in the multi-layered graphs (accessible at http://cytoscape.h3africa.org) generated from hypothesis-driven seeds (Table 1).

**FIGURE 5:**
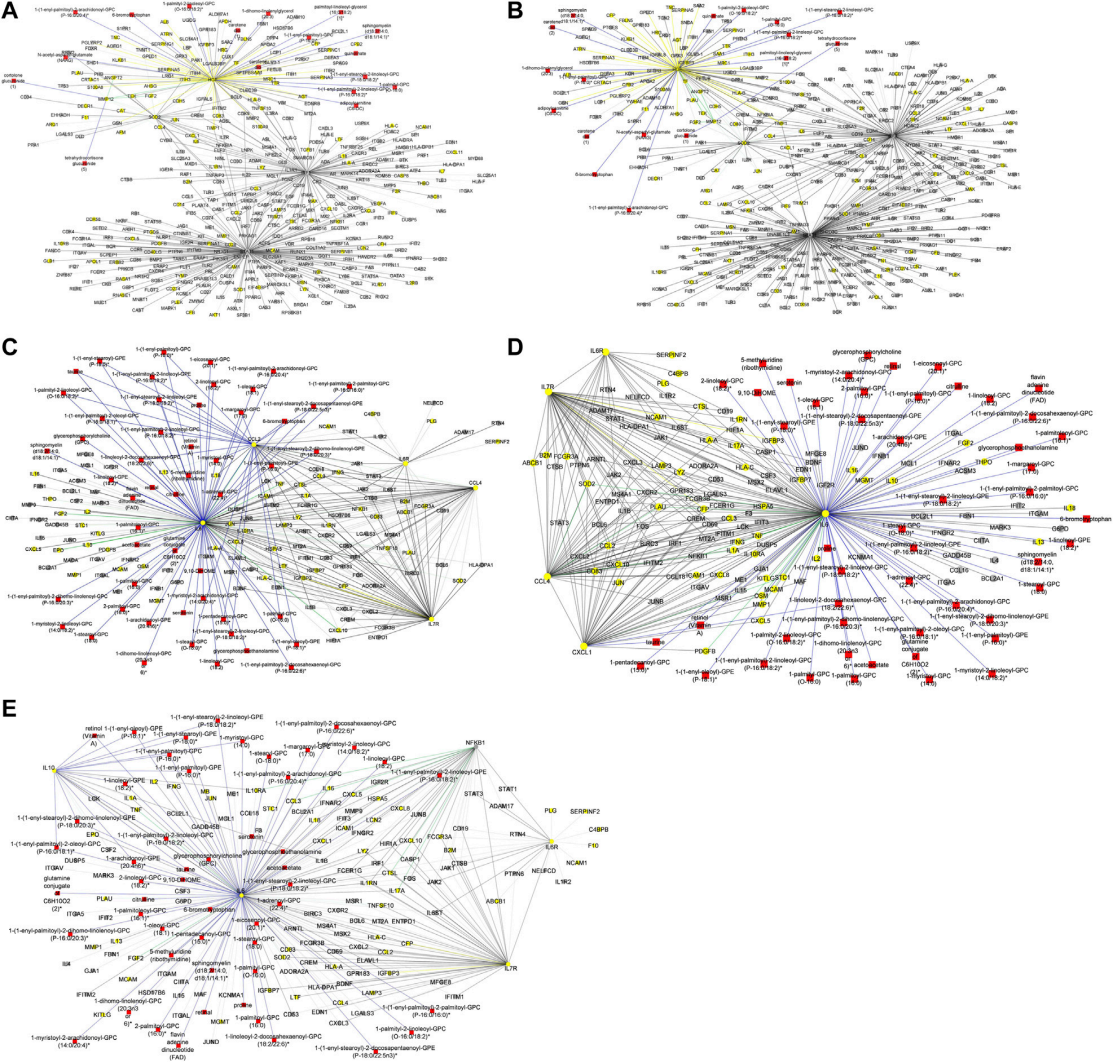
**(A)** Graph representation of subnetworks formed by hubs CCL2, CCL4, and IL7R, that establish direct interaction with seed nodes (IL6 and IL6R) as observed in the multi-layered graph generated for the mild disease state. The graph highlights the interaction of these hubs with other molecular features including proteins (yellow nodes), transcripts (grey nodes), and metabolites (red nodes). **(B)** Graph representation of subnetworks formed by hubs IL10, IL7R, and NFKB1, that establish direct interaction with seed nodes (IL6 and IL6R) as observed in the multi-layered graph generated for the moderate disease state. The graph highlights the interaction of these hubs with other molecular features including proteins (yellow nodes), transcripts (grey nodes), and metabolites (red nodes). **(C)** Graph representation of subnetwork formed by suberoylcarnitine metabolite and the cross-layer interaction with seed nodes (IL6 and IL6R), NFKB1, IL7R, and IL10 hubs. **(D)** Graph representation of subnetworks formed by hubs IL7R, CCL4, and CXCL1, that establish direct interaction with seed nodes (IL6 and IL6R) as observed in the multi-layered graph generated for the severe disease state. The blue edges represent protein-metabolite and transcript-metabolite interactions, the green edges represent protein-transcript interactions, the grey edges represent transcript-transcript interactions and, the yellow edges represent protein-protein interaction. **(E)** Graph representation of subnetwork formed by sphingomyelin (d18:2/21:0, d16:2/23:0) and the cross-layer interaction with seed nodes, IL6 and IL6R, and hubs IL7R, CCL4, IL6R, and CXCL1 as observed in the multi-layered graph generated for the severe disease state. The grey edges represent transcript-transcript interactions, the yellow edges represent protein-protein interactions, the cyan edges represent metabolite-metabolite interactions, the green edges represent protein-transcript interactions and the blue edges represent both protein-metabolite and transcript-metabolite interactions. Compounds for which a matching pure standard was not available for confirmation are denoted by adding an asterisk (*) symbol after the name of the compound.

To begin with, we evaluated the topology of the generated multi-layered graphs. Analysis of the generated multi-layered graphs revealed subnetworks established beyond those formed by the seed nodes, *IL6* and *IL6R*. Specifically, analysis of the multi-layered graph generated for the mild disease state revealed additional subnetworks, beyond those formed by the seed nodes (*IL6* and *IL6R*), involving hubs *CCL2, CCL4*, and *IL7R* (Figure 5A). These hubs establish direct interactions with the seed nodes and interactions with other molecular features, including interleukins (e.g., *IL13, IL18, IL1A*), and chemokines (e.g., *CCL3, CCL7, CXCL10*). Notable cross-layer interactions for the mild disease state were between *IL6* and *CCL2* with metabolites involved in various metabolic and inflammatory processes. These include but not limited to, taurine, 1-(1-enyl-palmitoyl)-2-oleoyl-GPC, 1-(1-enyl-palmitoyl)-GPC, 1-(1-enyl-palmitoyl)-GPE, 1-(1-enyl-stearoyl)-2-dihomo-linolenoyl-GPE, proline, 1-(1-enyl-palmitoyl)-GPC, 6-bromotryptophan, 1-margaroyl-GPC, and 1-myristoyl-GPC. This observation may further suggest a more efficient immune response to the virus among patients with mild disease. Specifically, 6-bromotryptophan is a biomarker indicative of COVID-19 severity, which is a finding concurrently echoed in the study by (Krishnan et al., 2021). The 6-bromotryptophan possess antiviral properties that can inhibit viral replication. Moreover, such brominated compounds are understood to exert influence on the immune system, particularly by modulating the kynurenine pathway of tryptophan metabolism, which is involved in regulating immune responses (Krishnan et al., 2021; Cihan et al., 2022; Takeshita and Yamamoto, 2022).

Our analysis of the multi-layered graph generated for the moderate disease state revealed new subnetworks formed by hubs *IL10*, *IL7R*, and Nuclear Factor Kappa B Subunit 1 (*NFKB1*). These hubs directly interact with the seed nodes (*IL6* and *IL6R*), but also with chemokines (e.g., *CCL4, CXCL5, CXCL8*) and other biosignatures such as *HLA-C, TNF, IFNG*, and *IFNAR2*, indicating a more complex interplay of immune cell recruitment and activation (Figure 5B). We also observed cross-layer interactions between *IL10* and *IL6*, and their connections to specific metabolites. Particularly, the cross-layer interactions involving *IL10* indicate a potential link between the immune and metabolic responses in moderate disease, suggesting a potential shift in the immune response dynamics in moderate cases as compared to mild cases. These metabolites including phosphatidylethanolamine, taurine, serotonin, and various GPC/GPE molecules such as 1-(1-enyl-palmitoyl)-2-oleoyl-GPC (P-16:0/18:1)*, 1-(1-enyl-palmitoyl)-GPC (P-16:0)*, 1-(1-enyl-palmitoyl)-GPE (P-16:0)*, 1-(1-enyl-stearoyl)-2-dihomo-linolenoyl-GPE (P-18:0/20:3)*, and 1-(1-enyl-stearoyl)-GPE (P-18:0)*, play crucial roles in both metabolic and inflammatory processes. Also, the *NFKB1* and *IL7R* subnetwork primarily interact with other proteins and transcripts. This suggests a focus on regulating gene expression and protein function, possibly contributing to the more severe inflammatory response associated with moderate disease. Compared to the multi-layered graph generated for the mild disease state, analysis of the multi-layered graph generated for the moderate disease state revealed a subnetwork formed by suberoylcarnitine (C8-DC) metabolite that established indirect cross-layer associations with the seed nodes and other hubs. Specifically, the suberoylcarnitine (C8-DC), plays a central role, influencing hubs like *IL6R, NFKB1, IL7R,* and *IL10* indirectly through its connection with *IL6* via a glutamine conjugate acting as a mediator (Figure 5C). This cross-layer pattern suggests a more nuanced control of immune and inflammatory processes compared to the mild disease state.

Analysis of the severe disease state multi-layered graph revealed subnetworks centered on *IL7R*, *CCL4*, and *CXCL1* (Figure 5D). These subnetworks exhibit significant cross-layer interactions with both protein-coding transcripts and metabolites, suggesting a complex interplay between gene expression and metabolic processes in this critical disease state. Similar to the cross-layer pattern observed in the moderate disease state, the severe disease network showcases another example of indirect cross-layer interaction via metabolites. Here, sphingomyelin (d18:2/21:0, d16:2/23:0) takes centre stage, influencing *IL6* and subsequently impacting *IL7R, CCL4, IL6R*, and *CXCL1* (Figure 5E). This further emphasizes the shift towards more nuanced, metabolite-driven cross-layer interactions in the severe disease state, potentially indicating immune-metabolic dysregulation in moderate and severe disease states.

Research suggests a correlation between *IL6* levels and altered metabolite levels including amino acids, fatty acids, and lipids among severe COVID-19 patients (Shen et al., 2020; Thomas et al., 2020). Similarly, *IL10* levels show various correlations with altered metabolite levels in infected COVID-19 patients (Shen et al., 2020). For instance, the study found that in COVID-19 patients, *IL10* levels were positively correlated with metabolites involved in glycolysis and the pentose phosphate pathway, such as glucose, fructose, and ribose-5-phosphate. The study also found a negative correlation between *IL10* levels and metabolites involved in the tricarboxylic acid cycle (TCA cycle) and oxidative phosphorylation, such as citrate, succinate, and ATP. These findings suggest that *IL10* may be associated with a shift in cellular metabolism towards glycolysis and away from oxidative phosphorylation in COVID-19 patients (Shen et al., 2020). The overall results highlight not only the influence of *IL6* in COVID-19 but also suggest that cross-layer interactions involving *IL6* influence clinical heterogeneity, thus influencing the dynamics of disease severity.

Furthermore, we evaluated the pairwise interactions and identified 807 interactions associated with one disease state of which approximately 37%, 37%, 2%, 15%, and 9% are transcript-transcript, lipid-lipid, protein-protein, metabolite-metabolite, and protein-metabolite interactions respectively (Supplementary File S2). Also, of these interactions, approximately 20%, 40%, and 40% are associated with mild, moderate, and severe disease states, respectively. Additionally, the analysis of interactions involved with only two disease states revealed 335 interactions, out of which approximately 6%, 85%, 7%, and 2% are protein-protein, transcript-transcript, lipid-lipid, and metabolite-metabolite interactions, respectively (Supplementary File S2). Of these interactions, approximately 16%, 10%, and 74% are involved with moderate-severe, mild-moderate, and mild-severe disease states, respectively.

Finally, we identified 894 interactions common across the three disease states, of which approximately 66%, 3%, 14%, and 17% are transcript-transcript, protein-protein, protein-transcript, and protein-metabolite interactions respectively (Supplementary File S2).

#### Characterizing multi-layered graphs

The observation that cross-layer interactions appear to be a distinctive factor for moderate and severe disease states using both data- and hypothesis-driven methods necessitated the determination of network statistics to further characterize the multi-layered graphs.

According to network density, network heterogeneity, and characteristic path length statistical metrics, graphs with high characteristic route length values have high network heterogeneity and comparatively low network density (Supplementary Data). The network statistical analysis (Table 4) further supported the idea that cross-layer interactions could be a factor underlying heterogeneity in disease severity among patients.

**TABLE 4:** Results from the statistical analysis of generated multi-layered graphs.

Statistical measure	Data-driven			Hypothesis-driven			Data-driven validation			Hypothesis-driven validation		
	Mild	Moderate	Severe	Mild	Moderate	Severe	Mild	Moderate	Severe	Mild	Moderate	Severe
Network density	0.008	0.006	0.006	0.010	0.007	0.008	0.102	0.050	0.027	0.049	0.045	0.020
Network heterogeneity	5.133	5.479	5.358	4.475	4.726	4.665	1.844	2.119	3.001	3.083	2.273	3.268
Characteristic path length	2.620	2.845	2.857	2.490	3.027	2.616	1.919	2.353	2.401	1.951	2.452	2.566

### Identifying disease states biosignature

#### Biosignatures discriminating between disease states based on data-driven seeds

The results of the multi-layer analysis (Supplementary File S2) formed the basis for identifying features that discriminate between disease states. Specifically, we explored the pairwise relations associated with one disease state (Supplementary File S2), as determined using the data-driven approach, and identified 173 discriminatory features (Table 5). Of note, the features identified are likely involved in all disease and non-disease phases because they form part of the biological system. However, identifying these features to discriminate between disease states in our analysis may suggest that they are differentially associated (either up or downregulated) in specific disease states.

**TABLE 5:** Identified biosignatures that discriminate disease states based on random walk with restart analysis using data-driven seeds.

Feature	Feature type	Disease state	Feature	Feature type	Disease state	Feature	Feature type	Disease state	Feature	Feature type	Disease state
*IRF8*	transcript	Severe	*CD83*	transcript	Mild	*IL1B*	transcript	Mild	*CCL18*	transcript	Mild
*KDR*	transcript	Severe	*CD8A*	transcript	Mild	*IL1RN*	transcript	Mild	*CCL2*	protein	Mild
*MMP9*	transcript	Severe	*CDH5*	transcript	Mild	*IL5*	transcript	Mild	*CCL20*	transcript	Mild
*PRTN3*	transcript	Severe	*CPB2*	protein	Mild	*RELA*	transcript	Mild	*CCL3*	transcript	Mild
*PTGES*	transcript	Severe	*CREM*	transcript	Mild	*IL7R*	transcript	Mild	*CCL5*	transcript	Mild
*YWHAE*	transcript	Severe	*CTSB*	transcript	Mild	*IRF9*	transcript	Mild	*CCL7*	protein	Mild
*CD34*	transcript	Moderate	*CTSC*	transcript	Mild	*JAK2*	transcript	Mild	*CCR1*	transcript	Mild
*ENPEP*	transcript	Moderate	*CTSL*	transcript	Mild	*JUN*	transcript	Mild	*CD14*	transcript	Mild
*MAPK8*	transcript	Moderate	*CXCL1*	transcript	Mild	*JUNB*	transcript	Mild	*CD163*	transcript	Mild
*NKRF*	transcript	Moderate	*CXCL10*	transcript	Mild	*KRT10*	transcript	Mild	*SERPINA4*	protein	Mild
*NLRP1*	transcript	Moderate	*CXCL11*	protein	Mild	*LCK*	transcript	Mild	*SERPING1*	protein	Mild
*PDGFB*	transcript	Moderate	*CXCL2*	transcript	Mild	*LGALS3*	transcript	Mild	*SH2D3C*	transcript	Mild
*SHC1*	transcript	Moderate	*CXCL8*	transcript	Mild	*TLR2*	transcript	Mild	*SIK1*	transcript	Mild
*SLC14A1*	transcript	Moderate	*CXCL9*	transcript	Mild	*TLR4*	transcript	Mild	*SLC3A2*	transcript	Mild
*YARS1*	transcript	Moderate	*CXCR2*	transcript	Mild	*TLR9*	transcript	Mild	*SCD*	transcript	Mild
*ABCB1*	transcript	Mild	*CXCR4*	transcript	Mild	*TNF*	transcript	Mild	*CYTIP*	transcript	Mild
*ADORA2A*	transcript	Mild	*CXCR6*	transcript	Mild	*TNFAIP3*	transcript	Mild	*DES*	transcript	Mild
*ADSL*	transcript	Mild	*CYBB*	transcript	Mild	*TNFSF10*	transcript	Mild	*DUSP5*	transcript	Mild
*AFM*	protein	Mild	*HNF4A*	transcript	Mild	*TPI1*	protein	Mild	*ENTPD1*	transcript	Mild
*APOE*	transcript	Mild	*HP*	transcript	Mild	*UBE2L6*	transcript	Mild	*EPCAM*	transcript	Mild
*ARNTL*	transcript	Mild	*HSD17B6*	transcript	Mild	*TGFB1*	transcript	Mild	*F3*	transcript	Mild
*ARRB2*	transcript	Mild	*HSPA5*	transcript	Mild	*STAT3*	transcript	Mild	*FCER1G*	transcript	Mild
*ASGR2*	transcript	Mild	*ICAM1*	transcript	Mild	*TAP1*	transcript	Mild	*FCGR3A*	transcript	Mild
*B2M*	transcript	Mild	*IFIT1*	transcript	Mild	*SERPINA1*	transcript	Mild	*FCGR3B*	transcript	Mild
*CD2*	transcript	Mild	*IFIT3*	transcript	Mild	*BAAT*	transcript	Mild	*FLNB*	transcript	Mild
*CD3D*	transcript	Mild	*IFITM2*	transcript	Mild	*BCL6*	transcript	Mild	*GPR183*	transcript	Mild
*CD4*	transcript	Mild	*IFNG*	transcript	Mild	*BDNF*	transcript	Mild	*GZMA*	transcript	Mild
*CD53*	transcript	Mild	*IGF1R*	transcript	Mild	*BIRC3*	transcript	Mild	*GZMB*	transcript	Mild
*CD69*	transcript	Mild	*IL10RA*	transcript	Mild	*BTK*	transcript	Mild	*HLA-DPA1*	transcript	Mild
*CD74*	transcript	Mild	*IL1A*	transcript	Mild	*CA1*	protein	Mild	*HLA-DPB1*	transcript	Mild
*HLA-DRA*	transcript	Mild	*MS4A1*	transcript	Mild	Unknown_mz_882.75427_+_RT_27.81	lipid	Mild	Unknown_mz_786.66003_+_RT_26.317	lipid	Mild
*HLA-E*	transcript	Mild	*MT2A*	transcript	Mild	Unknown_mz_921.69031_+_RT_29.122	lipid	Mild	Unknown_mz_786.66064_+_RT_27.722	lipid	Mild
*HLA-F*	transcript	Mild	*MYD88*	transcript	Mild	Unknown_mz_579.40155_+_RT_20.352	lipid	Mild	Unknown_mz_795.45471_+_RT_25.542	lipid	Mild
*S100A12*	transcript	Mild	*NFKB1*	transcript	Mild	Unknown_mz_600.52008_+_RT_23.106	lipid	Mild	Unknown_mz_806.62952_+_RT_25.891	lipid	Mild
*S100A8*	transcript	Mild	*NFKBIA*	transcript	Mild	Unknown_mz_650.53461_+_RT_24.007	lipid	Mild	Unknown_mz_810.66071_+_RT_27.059	lipid	Mild
*S100A9*	transcript	Mild	*NLRP3*	transcript	Mild	Unknown_mz_652.55096_+_RT_24.633	lipid	Mild	Unknown_mz_812.67621_+_RT_27.815	lipid	Mild
*LY6E*	transcript	Mild	*OASL*	transcript	Mild	Unknown_mz_702.56604_+_RT_23.89	lipid	Mild	Unknown_mz_817.63153_+_RT_27.802	lipid	Mild
*LYZ*	transcript	Mild	*PDCD1*	transcript	Mild	Unknown_mz_702.56689_+_RT_24.783	lipid	Mild	Unknown_mz_830.72375_+_RT_26.958	lipid	Mild
*MAPK14*	transcript	Mild	*PF4*	transcript	Mild	Unknown_mz_728.58246_+_RT_25.181	lipid	Mild	Unknown_mz_832.6452_+_RT_26.266	lipid	Mild
*MAPK3*	transcript	Mild	*PLAU*	transcript	Mild	Unknown_mz_736.64563_+_RT_24.633	lipid	Mild	Unknown_mz_836.67639_+_RT_27.461	lipid	Mild
*MB*	transcript	Mild	*PRF1*	transcript	Mild	Unknown_mz_750.66119_+_RT_25.544	lipid	Mild	Unknown_mz_838.69214_+_RT_27.059	lipid	Mild
*MEFV*	transcript	Mild	*PTPN6*	transcript	Mild	Unknown_mz_756.61407_+_RT_25.816	lipid	Mild	Unknown_mz_838.69214_+_RT_28.195	lipid	Mild
*MRC1*	transcript	Mild	*RAF1*	transcript	Mild	Unknown_mz_782.62958_+_RT_26.163	lipid	Mild	Unknown_mz_858.6604_+_RT_26.657	lipid	Mild

Of the 158 discriminatory features that were differentially associated with the mild disease state, approximately 78%, 6%, and 16% were transcripts, proteins, and (uncharacterized) lipids respectively. We identified chemokines (e.g., *CXCL10, CXCL12, CXCL5, CXCL8*, *CCL3, CCL8*), T-cell receptors (e.g., *CD38, CD40, CD48, CD68*), *HLAs* (e.g., *HLA-DPA1, HLA-DPB1, HLA-DRA, HLA-E, HLA-F*), interferons (e.g., *IFIT1, IFIT3, IFITM2*), and Toll-like receptors (e.g., *TLR2, TLR4, TLR9*) to discriminate the mild disease states. These features are involved in immune responses and play a part in viral entry into host T-cells (Del Valle et al., 2020; Lucas et al., 2020; Zayet et al., 2020; Shirato and Kizaki, 2021). For instance, *TLR2* activation increased the expression of *ACE2*, the receptor that SARS-CoV-2 uses to enter cells, suggesting that *TLR2* may play a role in viral entry into host T-cells. *IFIT1* has been shown to have antiviral activity against SARS-CoV-2, and may thus be an important component of the body’s immune response against the virus (Lucas et al., 2020). An elevated level of *CCL18* is associated with inflammation in the lungs of COVID-19 patients through the recruitment and activation of immune cells, including T-cells and dendritic cells in the lungs (Liao et al., 2020). Importantly, biosignatures including but not limited to HLA class I alleles, and *CXCL12*, have been validated through sequencing and cohort screening techniques to play a relevant role in immune defense against SARS-CoV-2 (Weiner et al., 2021; Martínez-Fleta et al., 2022).

Only 9 and 6 transcripts were associated with moderate and severe disease states respectively: no other omics features were identified to be associated with either of these disease states. There were also no metabolites that were differentially associated with any one of the disease states. This indicated that transcripts strongly differentiate the mild disease state from the moderate and severe disease states.

#### Biosignatures discriminating between disease states based on hypothesis-driven seeds

We explored the pairwise relations associated with one disease state (Supplementary File S2) based on analysis using seeds that were selected to test specific hypotheses. The results (Supplementary Table S3) revealed more biosignatures to be differentially associated with moderate and severe disease states than in the mild disease state. Additionally, unlike with the data-driven seed analysis, we observed more proteins and metabolites that discriminated between the moderate disease state than the others. Compounds for which a matching pure standard was not available for confirmation are denoted by adding an asterisk (*) symbol after the name of the compound.

We identified chemokines (*CXCL2, CXCL3, CXCR1, CXCR2, CXCR3, CXCR6*), cytokines (e.g., *TNF, TNFRSF1A, TNFSF10*), and other transcripts and proteins (e.g., *ATP6AP2*) that promotes the cytokine storm to discriminate the severe disease state from the other states. Concordantly, several studies have reported elevated levels of these features in COVID-19 patients, particularly those with severe disease (Coperchini et al., 2020; Li et al., 2020; Robinson et al., 2020). Also, some of the biosignatures including but not limited to *TNF, IL10*, have been verified using multiplex biosensor techniques to be associated with COVID-19, and as such these biosignatures could serve as markers to monitor the disease development (Del Valle et al., 2020; Madhurantakam et al., 2023).

The results further revealed lysophosphatidylcholine (LysoPC), diacylglycerol (DG), and triglycerides (TG) to discriminate severe disease states. Several studies have suggested the possible differential association of these lipids with COVID-19 pathogenesis and disease severity (Dai et al., 2021; Hammoudeh et al., 2021; Sheshan et al., 2021; Theken et al., 2021).

We identified metabolites such as kynurenate (Lawler et al., 2021), which have been reported to play a role in the cytokine storm and immune response, to discriminate the moderate disease state from the other states.

Comparing the biosignatures discriminating disease states further supports the idea that transcripts, metabolites, lipids, and proteins collectively influence disease progression beyond the mild state. In addition, we identified features (e.g., *SLC14A1, Adipoylcarnitine*) for which no direct roles in influencing disease severity have previously been reported: further research may provide important insights into their roles in COVID-19 pathogenesis and whether they might be useful targets for therapeutic intervention. Overall, these findings suggest that the discriminatory features play a significant role in the immune response to COVID-19 and that targeting them and/or their associated signalling pathways may be a potential therapeutic approach (Robinson et al., 2020).

### Enrichment analysis reveals enriched processes and pathways

#### Enrichment analysis of biosignatures that discriminate disease states based on data-driven seeds

From the discriminating features identified using the data-driven approach, we performed enrichment analysis (see Materials and Methods) based on the disease states they are differentially associated with (Supplementary File S3). The biological processes associated with proteins that discriminate the mild disease state are given in Supplementary File S3 Table S1. With a focus on the top 25 enriched biological processes and pathways, proteins were involved with chemotaxis (neutrophil, granulocyte, eosinophil, monocyte), regulating cytokine responses, and cell migration. These findings align with evidence of the role of chemotaxis in the initial response of detecting and destroying infected cells by following a chemical gradient of cytokines, chemokines, and other signalling molecules that are released by these cells (Veras et al., 2020). Further, the proteins were enriched in chemokine-mediated and interleukin-mediated pathways (Supplementary File S3 Table S2). For instance, regulation of the complement cascade pathway is important for controlling the immune response and preventing tissue damage. Several regulators of the complement system, including complement factor H, complement factor I, and CD59, are downregulated by SARS-CoV-2, which may contribute to complement dysregulation during infection (Carvelli et al., 2020; Risitano et al., 2020).

Transcripts discriminating the moderate disease state were enriched in those involved in cellular responses to organic substances and the mitogen-activated protein kinase (MAPK) cascade (Supplementary File S3 Table S5) but were especially enriched in transcripts involved in receptor-mediated signalling pathways (Supplementary File S3 Table S6). The MAPK pathways play a crucial role in regulating a variety of cellular responses, including cell proliferation, differentiation apoptosis, and immune response to COVID-19 (Bouhaddou et al., 2020).

The severe disease state discriminating transcripts were enriched in those involved in regulating ion transmembrane transporter activity, cell differentiation (dendritic, myeloid leukocyte), apoptotic processes, and mitochondrion organization (Supplementary File S3 Table S7) and enriched in signalling-related and regulatory-related pathways (Supplementary File S3 Table S8). The identified processes further align with the role of cell differentiation in COVID-19. For instance, dendritic cells act as sentinels to detect and respond to viral infections. They play a critical role in presenting viral antigens to T-cells, which, in turn, activate the immune response. During SARS-CoV-2 infections, dendritic cells can become infected which can lead to impaired antigen presentation and reduced activation of T-cells (Law et al., 2005). Also, myeloid leukocytes, including monocytes and macrophages, are important in the early immune responses to COVID-19. These cells can phagocytose viral particles and present viral antigens to T-cells, activating the immune response (Merad and Martin, 2020). However, during severe infections, excessive activation of myeloid cells can lead to a cytokine storm (Zhou et al., 2020), a dangerous immune response that can cause severe tissue damage and organ failure.

In addition to transcripts, some lipids discriminated against the different disease states. We were, however, unable to perform enrichment analysis on these because all of the discriminating lipids are presently uncharacterized.

#### Enrichment analysis of biosignatures that discriminate disease states based on hypothesis-driven seeds

We repeated the enrichment analysis but with discriminatory features identified using hypothesis-driven seed selections (*IL6* and *IL6R*) (Supplementary File S4 Tables S1–S17). The proteins and transcripts discriminating the mild, moderate, and severe disease states, for example, *IL13, CCL2*, *IL1A, SYK, IFNG, IL16, HMGB1, and TLR3* are involved in cytokine-, regulatory-mediated and apoptotic biological processes (Supplementary File S4 Tables S1, S3, S7, S9, S14, S17) and were also enriched in pathways including interleukin-mediated signalling, cytokine-mediated signalling, and cellular responses to stimuli (Supplementary File S4 Tables S2, S4, S8, S10, S15, S17). The fact that most of the biological processes and pathways are regulatory-, cytokine-, and cellular response-related agrees with other studies on disease severity. In the absence of appropriate regulatory T-cell activity to restrain the immune response to SARS-CoV-2 infections, the over-production of cytokines can ensue leading to a counter-productive cytokine storm (Moore and June 2020; André et al., 2022). Also, apoptotic biological processes are crucial in preventing severe disease by facilitating the death of infected cells to contain both the sizes of infection foci and the immune responses to the infected cells within these foci (Cizmecioglu et al., 2021; André et al., 2022).

The summary of metabolite pathways (Supplementary File S4 Table S5) linked to the mild disease states revealed metabolic processes that may play important roles in the pathophysiology of COVID-19 (Barberis et al., 2020; Song et al., 2020; Masoodi et al., 2022). For instance, sphingolipids are important components of cell membranes and are involved in a variety of cellular processes, including inflammation and apoptosis and the metabolism of these lipids has been implicated in the pathogenesis of viral infections, including COVID-19 (Song et al., 2020; Khan et al., 2021). Also, the dysregulation of arginine biosynthesis and lysine degradation may play a role in the pathogenesis of COVID-19 by modulating the immune response because arginine and lysine are essential amino acids that are involved in many biological processes including immune function and protein synthesis (Masoodi et al., 2022). We identified Sphingolipid metabolism processes to be common across all disease states. It has been suggested that the virus hijacks sphingolipid metabolism and dysregulates the metabolism activities to promote its replication and to evade the host immune response, aligning with the involvement of these processes in the pathogenesis of severe disease patients (Supplementary File S4 Tables S5, S6, S12) (Khan et al., 2021). We also identified other pathways linked with metabolic pathways discriminating moderate and severe disease states including, but not limited to, phenylalanine, tyrosine, and tryptophan biosynthesis pathways, and the pentose phosphate pathway.

Given the inability to perform enrichment analysis for uncharacterized lipids discriminating mild disease states, the summary of lipid pathways (Supplementary File S4 Tables S11, S13) linked with moderate and severe disease states revealed processes that may play important roles in the pathophysiology of COVID-19. Autophagy is involved in several other biological processes, including antigen presentation, cell death, and immune regulation to maintain or restore homeostasis. Dysregulation of these processes has been implicated in the pathogenesis of various diseases including COVID-19 and has even been presented as a target for therapeutics (García-Pérez et al., 2020; Yang and Shen, 2020). Arachidonic acid metabolism is the pathway responsible for the production of various bioactive lipids, including prostaglandins, leukotrienes, and thromboxanes. Dysregulation of arachidonic acid metabolism has been implicated in the pathogenesis of numerous diseases and syndromes, including inflammation, cancer, and cardiovascular disease (Masoodi et al., 2022).

Lipids are central components of cell membranes, such that dysregulation of pathways such as glycosylphosphatidylinositol (GPI) anchor biosynthesis and glycerophospholipid metabolism occurred in all disease states (Supplementary File S4 Tables S11, S13). For instance, Glycosylphosphatidylinositol (GPI)-anchor biosynthesis—GPI-anchor biosynthesis is the process by which GPI-anchored proteins are synthesized and inserted into the plasma membrane. GPI-anchored proteins play critical roles in cell signalling, immune function, and development. Glycerophospholipid metabolism is the metabolic pathway responsible for the synthesis and degradation of glycerophospholipids, which are also essential components of cell membranes. Abnormalities in glycerophospholipid metabolism have been previously implicated in the pathogenesis of several diseases including COVID-19 (Li et al., 2022; Oliveira et al., 2022).

#### Comparing results from data-driven and hypothesis-driven approach

Although the methodologies differed, the findings from both data-driven and hypothesis-driven approaches have contributed to our understanding of COVID-19 disease progression. The data-driven approach was useful for uncovering unexpected findings and trends, particularly concerning *STAT1* and *SOD2* influence on disease progression thereby sparking new hypotheses and insights. However, the random walk analysis using both the data-driven and hypothesis-driven approaches yielded distinct multi-layered graphs (Figures 4, 5) characterized by different hubs and interactions, highlighting the unique perspectives offered by each method. With these differences, a consistent finding emerged from both approaches: cross-layer interactions between omics features play a role in disease state. Notably, both approaches revealed transcripts, especially cytokines and inflammatory biosignatures as key contributors to distinguishing disease states in both approaches. Also, both approaches revealed some overlapping biosignatures (as shown in Table 5; Supplementary Table S3).

#### Validating the integrative network-based multi-omics-driven data approach and replicating results from independent data

Random walk network analysis was performed on disease-state specific omics-graphs and specifically investigated the behaviour of a new multi-layer graph generated from different datasets from the perspective of network statistical parameters (Supplementary Data). As part of the analysis, published metabolomics, transcriptomics, and proteomics features reported to be associated with the various COVID-19 disease states were retrieved. Specifically, transcriptomics features specific to mild, moderate, and severe disease states were retrieved from (Alqutami et al., 2021). Proteomics features specific to the moderate disease state were retrieved from (Zhong et al., 2021). Features specific to the mild and severe disease states were retrieved from (Patel et al., 2021), and (Suhre et al., 2022). We generated protein-protein interaction networks using GeneMANIA. Metabolites differentially associated with mild, moderate, and severe COVID-19 disease states were retrieved from (Jia et al., 2022). For each disease state, by using the metabolite KEGG IDs as inputs we constructed a metabolite-metabolite interactome using MetaboAnalyst 5.0 (Pang et al., 2021), which is a knowledge-driven multi-omics integration platform (https://www.metaboanalyst.ca/). We further constructed the metabolite-protein interactome for each disease state from MetaboAnalyst 5.0 by using the metabolite KEGG IDs and gene IDs of the features that were differentially associated with the different disease states. We also included the lipid interactome generated from (Overmyer et al., 2020), datasets. We repeated the random walk analysis using the data-driven and hypothesis-driven seeds; except that, since hydroxyoctanoate was not a feature in the generated networks, it was excluded from the data-driven seeds. We also performed a statistical network analysis of the multi-layered graphs generated. We observed that network heterogeneity and characteristic path length metrics correlated with disease severity (Table 4). Following the analyses that we previously performed on our multi-layered graphs, we observed that network density decreased with disease severity. The statistical analysis also supported the observation during our multi-layered network analyses that crosstalk between features across multiple omics layers (layers containing different feature types) relates to disease severity and could be a distinctive factor underlying the heterogeneity in disease severity among patients.

## Discussion

Different single omics (Fraser et al., 2020; Alqutami et al., 2021; Daamen et al., 2021; Jain et al., 2021; Patel et al., 2021; Zhong et al., 2021; Ciccarelli et al., 2022; Jia et al., 2022; Páez-Franco et al., 2022; Roberts et al., 2022; Suhre et al., 2022) and multi-omics (Barh et al., 2020; Bernardes et al., 2020; Overmyer et al., 2020; Su et al., 2020; Stephenson et al., 2021; Sun et al., 2021; Suvarna et al., 2021; Chattopadhyay et al., 2022; Gygi et al., 2023) studies have been conducted to provide insights into the aetiology of COVID-19 disease severity. However, computational network-based integrative analysis that considers different omics profiles from multiple studies with existing biological knowledgebases to explore proteomics, transcriptomics, metabolomics, and lipidomics biosignatures and their connections across different disease phases, to help explain clinical heterogeneity is limited.

Here we have hypothesized that i) investigating biosignatures across COVID-19 disease phases would provide insights into the observed clinical heterogeneity and facilitate an understanding of factors associated with disease severity, and ii) associations between the biosignatures within a biological network would permit the prioritization of those biosignatures that discriminate the disease states, which may, in turn, provide insights into drug research. An integrative multi-omics network analysis was performed by using proteomics, transcriptomics, metabolomics, and lipidomics data from (Overmyer et al., 2020), and (Su et al., 2020). We demonstrate an approach for harmonizing the clinical severity of COVID-19 patients from independent studies leveraging the WOS and patient clinical metadata. In addition, through our workflow, both data-driven and hypothesis-driven approaches were leveraged in an interoperable way at different biological scales, providing an impact on our understanding of the disease phases. We believe that this approach forms the basis of classifying COVID-19 patients from independent multi-omics studies and allows for the grouping of omics experimental data into disease states to perform computational network-based integrative multi-source multi-omics analysis.

From the random walk network analysis on the disease-state specific omics-graphs, we noticed some unique patterns in the cross-layer interactions for mild, moderate, and severe disease states from both the hypothesis- and data-driven approaches. Specifically, random walk analysis using the hypothesis-driven seeds resulted in networks with both a greater variety of features (particularly metabolites and lipids) and more interactions between different feature types, than was achieved in networks generated using data-driven seeds. This observation might be partly attributable to the fact that *IL6* and *IL6R* (the two hypothesis-driven seeds that were used) have a profound role during the anti-inflammatory response to COVID-19. Compared to interactions established by *IL6R* as a seed, we observed that *IL6* as a seed established a network with more interactions between proteins, transcripts, and metabolites related to cell function and immune responses (accessible at http://cytoscape.h3africa.org). Whereas, *IL6R* as a seed yielded a network that primarily captured protein and transcript interactions. Notable among the metabolites interacting with *IL6* is taurine, an amino sulfonic acid involved in the regulation of oxidative stress, which is known to play an important role in COVID-19. Taurine levels have been reported to decrease in COVID-19 patients which potentially modulates disease progression via its antiviral, antioxidant, anti-inflammatory, and vascular-related effects (van Eijk et al., 2022). Other metabolites interacting with *IL6* included serotonin, a neurotransmitter, known to play important roles in the immune system and in regulating inflammatory responses (Eteraf-Oskouei and Najafi, 2022).

Similarly, cross-layer patterns were observed from the random walk analysis using the data-driven approach among *HGF, STAT1,* and *SOD2* (Figure 4). From the data-driven seeds (Table 1), *STAT1* established connections with hubs *IRF1*, and *CCL4* in mild, and hub *IRF1* in both moderate and severe disease states (Figure 4). *SOD2* connects with hub *IRF1* in the mild, moderate, and severe disease state networks (Figure 4). Also, 3-hydroxyoctanoate interacts with 3-hydroxyexanoate and 3-hydroxydecanoate in all disease states. The random walk analysis provided not only an insight into network connectivity but also the results from the network statistical analysis (Table 4) were consistent and overlapped. Of note, the outcome from the random walk is based on the choice of seeds (Table 1) used for the random walk analysis. The analysis generated a multi-layered graph for each disease state based on the exploration of the seeds across the disease-state specific omics-graphs.

Despite the heterogeneity of COVID-19 disease outcomes, the individual mild, moderate, and severe disease states seem to have characteristic degrees to which transcript, protein, metabolite, and lipid features associatively interact both with themselves and with one another. Upon evaluating the multi-layered graphs for the various disease states, we identified several associative interactions that were present irrespective of disease state and a number that seemed to be specific for particular disease states (Supplementary File S2). These associations should be further analysed to better understand the causal effects.

In general, we observed that transcript-transcript interactions were the most commonly detected across all the disease states whereas metabolite-metabolite and lipid-lipid interactions were least commonly detected. This observation could partly be attributed to the fact that between 4 and 16 times more individual transcript features are present in the transcriptomics experimental datasets than are present in the lipidome and metabolome datasets respectively. However, irrespective of these differences, we observed that major distinctions among disease states are a result of cross-layer interactions: with protein-metabolite interactions being particularly notable. Specifically, we observed an overall increase in cross-layer interaction with disease severity using both data-driven and hypothesis-driven seeds for the network exploration. We tested network statistics to confirm the network behaviour across disease states in both the original datasets and the validating datasets and found that cross-layer interactions within networks could be a distinctive feature of severe COVID-19.

From the evaluation of interactions associated with the disease states, we identified biosignatures of different omics types that discriminate specific disease states (Table 5; Supplementary Table S3). These biosignatures are differentially associated with the disease states (Overmyer et al., 2020; Su et al., 2020). Further, from the enrichment analysis of these discriminatory biosignatures (Supplementary Files S3, S4), we notice cytokine-, regulatory-mediated, and cellular responses to infection processes were apparent among disease-state discriminating transcripts and proteins identified from both the data-driven and the hypothesis-driven approach. This gives us a general overview that, despite the heterogeneity of COVID-19 disease outcomes, the biological processes and pathways underlying the disease could be related, but with varying expression levels of the biosignatures involved. As expected, we identified the metabolic processes related to the disease-discriminatory metabolites. However, other metabolite-related pathways relating to the degradation and/or synthesis of essential amino acids were noticeable among moderate and severe disease-state-discriminating metabolites (Supplementary File S4 Tables S5, S6, S12). Knowing that these essential amino acid processes contribute to protein synthesis, therefore, suggests that the disturbance of protein synthesis could contribute to the severity of the disease.

This study did not consider the different treatments received by the patients reported in (Su et al., 2020), and (Overmyer et al., 2020), as a study variable during the harmonization and analysis processes. This is partly attributed to the different treatment options used and the limited treatment information reported in the study, especially by (Su et al., 2020), thus making it a difficult study variable to consider. We prioritized the patients’ disease states by categorizing them based on the severity of their COVID-19 condition—whether mild, moderate, or severe. This classification, inclusive of patients with potential co-infections or commodifies, was maintained throughout the harmonization process and subsequent downstream analysis.

We acknowledge that both (Su et al., 2020), and (Overmyer et al., 2020), utilized different approaches in blood sample collection and processing thereof, which may have impacted the expression levels of the various multi-omics features. Also, the issue of different methods, instruments, or scales used to collect data could have contributed to heterogeneity among the datasets making data merging a non-trivial task. However, we performed data cleaning, implemented the normalization statistical method, and performed data harmonization on the multi-omics experimental datasets (as described in the methods section) before downstream analysis as a measure of controlling heterogeneity and facilitate data merging. In addition, we have combined the co-expression graphs from both studies, as a way of controlling the impact of the between-study methodological differences on the multi-omics feature expression levels as well as any bias introduced during the harmonization process. We only used lipidomics experimental data from a single study and it is likely therefore that this part of our analysis was proportionally underpowered relative to that involving transcripts, proteins, and metabolites. Furthermore, most of the lipids used for the analysis were uncharacterized and we were therefore unable to map to lipid names as well as perform enrichment analyses on them. In the multi-layered networks we created, we also did not discover any protein-lipid edge types. This may be attributable in part to the protein-lipid bipartite data and the seed exploration during random walk analysis as well as limited information on interaction involving annotated features and unannotated features. Furthermore, future investigations could consider incorporating not only characterized (annotated) lipids but also additional data types such as epigenomics, microbiomics, and immunomics. Moreover, from the analysis, we observed more transcript-transcript interactions as compared to other omics features. This observation is at least partly attributable both to the unevenness in the number of features measured in the different omics experiments (with the transcriptomics experiment examining between 5 and 100 times more features than other types of omics experiments) and the fact that more research efforts have been focused on transcriptomics analyses than on those of other omics types. Despite these limitations, our study has some obvious strengths, especially using the hypothesis-driven approach, we provide insight into the specific roles of *IL6* and *IL6R* in COVID-19 progression, providing targeted insights into this crucial pathway. On the other hand, the data-driven approach revealed connections between biosignatures, like *STAT1* and *SOD2*, highlighting their previously unknown influence on disease course and generating exciting new avenues for exploration. The results presented revealed biosignatures and their interactions related to disease severity. Having demonstrated that cross-layer interactions could be a distinctive feature, if not a hallmark, of severe COVID-19, it warrants deeper investigation into the potential causal relationships that these cross-layer interactions, have with disease progression: relationships that might illuminate ways to prevent and/or reverse this progression.

*IL6* is a vital innate immune cytokine in protection against other viral infections such as influenza A virus, which can cause pneumonia (Guirao et al., 2020). An increase in *IL6* levels has previously been observed in patients with respiratory dysfunction, implying a possible mechanism of cytokine-mediated lung damage caused by COVID-19 infection (Gou et al., 2020; Guirao et al., 2020). Our hypothesis-driven approach revealed that higher COVID-19 disease severity was associated with an increased number of interactions between *IL6* and other multi-omics layers, therefore suggesting that our approach may discriminate between COVID-19 and other respiratory disorders. However, to confirm we will need future studies on the evaluation of network-based multi-omics approaches for COVID-19 compared to other infectious diseases, including those of viral and bacterial nature.

Our study suggests a deeper understanding of the underlying biological interactions in different phases of COVID-19 disease. The results (Figures 4, 5, along with Table 5; Supplementary Table S3 and Supplementary Material S2) present an extensive analysis of multi-layered graphs generated by complementary approaches including data-driven and hypothesis-driven seeds, and shed light on the complex interactions underlying different phases of COVID-19. These findings suggest a nuanced understanding of how various molecular features interact and influence disease severity. However, heterogeneity, different sample sizes, and sensitivity of types of samples used for sequencing across studies might be a potential source of bias. The hypothesis-driven approach offers a reductionist strategy for experimental proof whereas the data-driven approach offers a holistic strategy and hypothesis generation. We recommend considering both data- and hypothesis-driven approaches in studies utilizing multiple source omics datasets. Also, the complexity of disease severity harmonization, identifier mapping, and feature selection might be potential sources of bias in our studies.

The multi-omics harmonization process and integration strategy implemented in this study can be applied to other infectious and complex diseases, thus contributing to aggregating data from multiple sources for downstream analysis. Importantly, the algorithmic framework implemented in this study can be translated to other diseases to investigate biosignatures that underly disease progression, and relevant drug targets, and to understand disease mechanisms from the perspective of different omics layers (Yan et al., 2018). For instance, the methods and algorithm from this study can be used to investigate the underlying biology of complex diseases such as cancer in the context of investigating cancer subtypes and identifying the omics alterations that could help discriminate tumors, thus leading to proper diagnostics and prognosis (Menyhárt and Győrffy, 2021). It is however important to consider a more comprehensive list of seeds to help interpret and extend the findings.

In the biomedical context, data integration across different omics layers may help detect biosignatures connecting genomic events to clinical factors (such as response to treatment, mRNA expression levels). This would help to predict the drivers of disease outcomes, eventually leading to better patient stratification that can be translated to better clinical tests, early intervention, and more efficient personalized therapies (Menyhárt and Győrffy, 2021).

## Conclusion

In this work, we delved into the identification and characterization of biosignatures and their specific molecular features that underly various phases of COVID-19 disease, by using an integrative and network-based approach to analyse multi-omics data. We emphasized the critical importance of integrating multi-omics data, to elucidate the molecular dynamics responsible for the wide-ranging clinical presentations of COVID-19. This integration considers both prior knowledgebases and multi-omics data from independent studies.

Our study not only pinpoints biosignatures that distinguish between disease states, but also demonstrates a correlation between the severity of disease states of COVID-19 and cross-layer interactions of proteins, transcripts, metabolites, and lipids.

We are confident that the presented multi-omics data harmonization and network-based analysis approach can also be applied to other diseases. To facilitate replication of our approach, we provide a containerized workflow with an expanded readme file at https://github.com/francis-agamah/Multi-source-multi-omics-network-analysis. All other data and its Supplementary Material files generated during this study are included in the github repository.

## Data Availability

The original contributions presented in the study are included in the article/Supplementary Material, further inquiries can be directed to the corresponding authors.

## Supplementary material

Online resource: We have hosted interactive networks on the website (http://cytoscape.h3africa.org). The graphs hosted on the website describe interactions between proteins (green nodes), transcripts (grey nodes), lipids (pink nodes), metabolites (cyan nodes), and seeds (yellow nodes) across mild, moderate, and severe COVID-19 states. The edges describe protein-protein interactions (green edges), lipid-lipid interactions (pink edges), transcript-transcript interactions (grey edges), metabolite-metabolite interactions (cyan edges), protein-metabolite (blue edges), transcript-metabolites (blue edges).
